# Buttermilk for Urticaria in Children

**Published:** 1909-07-17

**Authors:** 


### BUTTERMILK FOR URTICARIA IN CHILDREN.

Much is being written just now upon the subject of lactic acid bacilli in the treatment of gastro-intestinal diseases such as colitis; and there are many who hold that special preparations made by acting upon milk with particular cultures of micro-organisms—notably the Bulgarian bacillus—are essential to success. Whether or not these specialties are required in the treatment of some cases, there can be little doubt that ordinary buttermilk with its mixed micro-organisms is of great value in the cure of many minor ailments. We refer, of course, to fresh buttermilk, or buttermilk which is not so soured as to be absolutely difficult to swallow. Fresh buttermilk is very palatable, at any rate to adults and older children; and although it may have to be specially ordered, it is usually obtainable even in large towns, if the dairyman is asked to obtain a daily supply of it.

Skin eruptions in children are frequently due to gastro-intestinal troubles; urticaria is familiar in this respect, and even severe dermatitis is sometimes seen. A mixture containing salol, together with a mild laxative, may suffice to cure the condition sometimes; but when a case is met with in which the cure is less easily effected, possibly because of persistence of the intestinal cause, it may be worth while to prescribe buttermilk—a tumblerful or more during the day for an adult, less for a boy or girl. The results obtained have been excellent, and the cure has often been rapid and complete. When there has been diarrhœa and decomposition of the fæces the stools have become regular and have lost their fœtid smell; the intense irritation of the skin diminishes and then disappears. When there has been more than urticaria, a general eruption, which might be classified by some as a dermatitis, by others as an eczema, a cure has been obtained in periods varying between twelve days and four weeks. The buttermilk acts, apparently, by acclimatising harmless lactic acid bacilli inside the bowel, with consequent inhibition, or even cessation, of the growth of less harmless bacteria. There is no danger, and the treatment may be continued for a long time. It is, of course, a return to an old-time custom when buttermilk was freely drunk by those who lived in the country.

